# The Use of Tactile Sensors in Oral and Maxillofacial Surgery: An Overview

**DOI:** 10.3390/bioengineering10070765

**Published:** 2023-06-26

**Authors:** Pietro Navalesi, Calogero Maria Oddo, Glauco Chisci, Andrea Frosolini, Paolo Gennaro, Vincenzo Abbate, Domenico Prattichizzo, Guido Gabriele

**Affiliations:** 1 The BioRobotics Institute, Scuola Superiore Sant’Anna, 56127 Pisa, Italy; 2 Department of Information Engineering, Università di Pisa, 56127 Pisa, Italy; 3 Department of Excellence in Robotics & A.I., Scuola Superiore Sant’Anna, 56127 Pisa, Italy; 4 Interdisciplinary Research Center Health Science, Scuola Superiore Sant’Anna, 56127 Pisa, Italy; 5 Department of Medical Biotechnologies, School of Oral Surgery, University of Siena, 53100 Siena, Italy; 6 Maxillofacial Surgery Unit, Department of Medical Biotechnologies, University of Siena, 53100 Siena, Italy; 7 Head and Neck Section, Department of Neurosciences, Reproductive and Odontostomatological Science, Federico II University of Naples, 80013 Naples, Italy; 8 Department of Information Engineering and Mathematics, University of Siena, 53100 Siena, Italy

**Keywords:** haptic, sensors, sensorized tools, platform, oral, maxillofacial, surgery

## Abstract

Background: This overview aimed to characterize the type, development, and use of haptic technologies for maxillofacial surgical purposes. The work aim is to summarize and evaluate current advantages, drawbacks, and design choices of presented technologies for each field of application in order to address and promote future research as well as to provide a global view of the issue. Methods: Relevant manuscripts were searched electronically through Scopus, MEDLINE/PubMed, and Cochrane Library databases until 1 November 2022. Results: After analyzing the available literature, 31 articles regarding tactile sensors and interfaces, sensorized tools, haptic technologies, and integrated platforms in oral and maxillofacial surgery have been included. Moreover, a quality rating is provided for each article following appropriate evaluation metrics. Discussion: Many efforts have been made to overcome the technological limits of computed assistant diagnosis, surgery, and teaching. Nonetheless, a research gap is evident between dental/maxillofacial surgery and other specialties such as endovascular, laparoscopic, and microsurgery; especially for what concerns electrical and optical-based sensors for instrumented tools and sensorized tools for contact forces detection. The application of existing technologies is mainly focused on digital simulation purposes, and the integration into Computer Assisted Surgery (CAS) is far from being widely actuated. Virtual reality, increasingly adopted in various fields of surgery (e.g., sino-nasal, traumatology, implantology) showed interesting results and has the potential to revolutionize teaching and learning. A major concern regarding the actual state of the art is the absence of randomized control trials and the prevalence of case reports, retrospective cohorts, and experimental studies. Nonetheless, as the research is fast growing, we can expect to see many developments be incorporated into maxillofacial surgery practice, after adequate evaluation by the scientific community.

## 1. Introduction

Computer Assisted Surgery (CAS) introduces several benefits in surgical practice and training: improving surgeon’s precision, repeatability, and dexterity, simultaneously providing hand-tremor filtration and motion scaling [1,2]. On the other hand, technological limitations include high cost, variable learning curve, and long setup times. In particular maxillofacial surgery requires high accuracy, stability, and transversal skills ranging from dentistry to general medicine [3]. Robot-assisted systems employed in dental and maxillofacial surgery could reduce risks for patients allowing some advantages such as improving the position and orientation of surgical tools, prevention of injuries to nerves and vessels in neck/head region avoiding accidental damages due to human factors at the same time.

For years, many efforts have been made to overcome the technological limits of CAS, the main one is the complete lack of haptic/force feedback to the surgeon [4]. Haptic feedback requires ad hoc tactile sensors to acquire haptic information, tools able to integrate them, and interfaces to display information or let the user feel sensations [5,6]. Tactile and force feedback are desirable to avoid injuries due to excessive applied forces when the tool–tissue interactions fall below the human sensory threshold [1], to facilitate novices’ learning while performing a complex or new task, to improve soft tissues/membranes manipulation and restore natural haptic feedback lost because the surgeon cannot manipulate the instruments directly [7]. It has been demonstrated that a shortage of haptic feedback in commercial surgical tools and platforms leads to a decrease in surgeons’ skills [4]. Therefore, the research has pointed to novel tactile sensors and sensorized tools development able to be integrated into commercial existing surgical platforms [1]. Some authors reported potential benefits and limits of haptic feedback in robot-assisted minimally invasive surgery (RMIS) as well as in head and neck surgery [4,7,8] and agreed that commercial existing systems are not able to provide adequate haptic sensations. The literature is not completely one-sided in evaluating haptic feedback essential in CAS systems [9,10] with the assumption that the surgeon’s experience and visual feedback alone can balance the lack of haptic feedback in RMIS practice. However, the effectiveness and necessity of integrated haptic feedback are proved in fields of application such as retinal and ENT microsurgery [11,12], endovascular treatments [13], neurosurgery, and magnetic resonance imaging (MRI)-guided interventions [14]. The major shortage of studies can be noticed in the dental and maxillofacial surgery field, with only partially consistent literature covering haptic digital simulators and training platforms and a paucity of specifically designed tactile sensors or tools. Basically, novel design tactile sensors for biomedical purposes should be “skin-like” with high sensitivity to measure 3D small forces (in the range 0.01–10 N), fast response (less than 1 ms), and high spatial resolution (1 mm for sensitive sites) with multimodal sensing capabilities. Tactile transduction mechanisms, able to convert tactile stimuli into electrical signals, can be employed in electrical-based or optical-based technologies. Electrical-based sensors (piezoresistive, piezoelectric, capacitive, inductive sensors) and optical-based sensors have different peculiarities and drawbacks, described in detail in Section 3.1. Their usage depends on different factors such as the anatomical district of employment, costs, and environmental elements.

The aim of the present work is to provide a comprehensive overview of different tactile sensors and haptic technologies especially designed for dental and maxillofacial surgery and telemedicine. Furthermore, current limits and future progress will be discussed.

The studies have been categorized into four different areas based on the presented technologies with a specific focus on maxillofacial/dental applications:

- Tactile sensors for instrumented tools;
- Sensorized tools for contact forces detection;
- Haptic interfaces and main actuation principles for remote tactile feedback;
- Integrated platforms for surgery and telemedicine.

In particular, these four areas are strictly close to each other. Every category involves fundamental technologies to restore the sense of touch in CAS. Tactile sensors—integrated into sensorized surgical tools—detect contact forces and stimuli, while adequate haptic devices supply the feedback to the surgeon. A surgical robotic platform is a two-way system: the user maneuvers the haptic device and commands are sent to the robotic end effector; meanwhile, the tool–tissue contact forces are detected with force/torque sensors and sent back to the haptic device.

## 2. Materials and Methods

The study protocol was defined under the Preferred Reporting Items for Systematic Reviews and Meta-analyses (PRISMA) statement, as previously reported [15]. The questions were formulated based on the PICO model [16], and record search and study selection strategies were developed. The research question focused on the study, research and/or application of haptic sensors, sensorized tools, tactile sensors, and integrated platforms for dentistry and maxillofacial surgery. Manuscripts were searched electronically through Scopus, MEDLINE/PubMed, and Cochrane Library databases by two independent reviewers (G.C., A.F.) without date restriction until 1 November 2022, using keywords and Boolean operators as follows: (“haptic” OR “sensorized” OR “tactile” OR “integrated”) AND (“sensors” OR “tools” OR “platform” AND “dental” OR “dentistry” OR “maxillofacial”). The “Related articles” option on the PubMed homepage was also considered. No filters were applied. Collected citations were recorded, duplicates were eliminated via the reference management tool EndnoteTM (Clarivate), and the remaining were screened by the three independent reviewers (FDS, FDA, MPDP), who then screened relevant abstracts. The full texts of these potentially eligible title-abstracts were obtained, contacting the study authors if full texts were unavailable, and were independently reviewed by the same authors (G.C., A.F.). Any disagreements were clarified by consultation, and in case of doubt, another author (G.G.) was consulted. The identified full texts were screened for original data and the related references were retrieved and checked manually for other relevant studies. Only relevant articles according to the research question were included; non-English language studies were excluded. Data were extracted independently and in duplicate by two authors (G.C., A.F.) using a standardized data extraction form developed based on the models recommended for intervention reviews of RCTs and non-RCTs before data extraction [16]; a third author (G.G.) was involved in case of disagreement. Moreover, the grading of each included study was carried out by two different authors (A.F. and G.C.), and any disagreement was solved by a discussion with a third author (G.G.). The appropriate JBI’s Clinical Appraisal Tool for each different type of included study was applied. A scale for the quality assessment of narrative review articles (SANRA) was used for the review articles included.

## 3. Results

**Figure 1:**
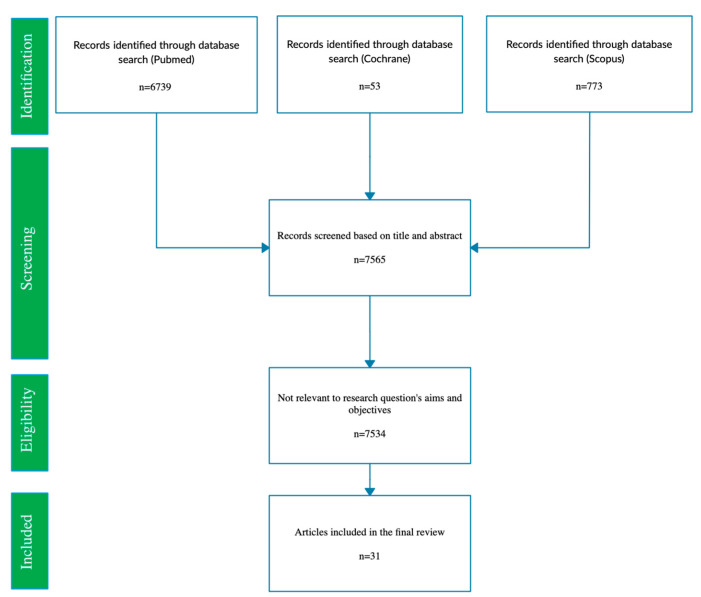
PRISMA diagram resembling article selection process from identification to inclusion.

After searching electronic databases, 7565 original manuscripts were retrieved as shown in Figure 1. After applying inclusion/exclusion criteria, 31 articles were included in the present overview [17,18,19,20,21,22,23,24,25,26,27,28,29,30,31,32,33,34,35,36,37,38,39,40,41,42,43,44,45,46,47]. The included articles were mostly experimental studies [22,24,26,27,28,29,30,32,33,34,38,40,43,44,45,46,47], reviews [17,18,19,20,21,25,41,42], and a few clinical studies mainly consisting of case reports [23,27,31,39,45] and one retrospective study [36]. Grading of each included study was performed by two different authors (A.F. and G.C.), any disagreement was solved by a discussion with a third author (G.G.) (Table 1, Table 2, Table 3 and Table 4).

### 3.1. Tactile Sensors for Instrumented Tools: Dentistry and Maxillofacial Applications

The transduction mechanisms of tactile sensors can be divided into two different categories: electrical-based sensors (piezoresistive, piezoelectric, capacitive, and inductive sensors) and optical-based sensors [17,18]. Capacitive and inductive tactile sensors display changes in capacitance and inductance, respectively. They are actually hardly used in the biomedical scene because of the weaknesses listed in Table 5, although the future potentialities of capacitive sensors, especially in MIS. Piezoresistors show resistivity changes as a consequence of an applied force. Advantages include high spatial resolution and sensitivity while the main weaknesses are low frequency response and repeatability [17]. Strain gauge sensors consist of a resistive elastic unit and their sensing principle is based on resistance changes with strain because of shape deformation [19]. Resistance variation is a function of the applied strain. Advantages include ease of design and high spatial resolution while the main handicaps are large hysteresis and non-linear response [20]. Piezoelectric tactile sensors transduce force/pressure in a proportional voltage (the change of electrical polarization of the piezoelectric material’s element induces a mechanical deformation), but high accuracy is balanced by low spatial resolution [20]. The optical ones, including optical fiber and Fiber Bragg Grating (FBG) sensors, obtain tactile information by analyzing wavelength changes [20,21]. An FBG sensor is a sensing element embedded into an optical fiber. FBG sensors detect force/torque data using the variation in the FBG reflection spectrum induced by the applied load to be measured. A multicomponent FBG-based sensor should measure force/torque components along the x-, y-, and z-axis. Optical sensors have unique properties essential in biomedical applications such as biocompatibility, chemical inertness, immunity to electromagnetic interference, and high sensitivity while main limits include susceptibility to temperature, relatively high cost, complex information processing system, and size limitations. As a consequence, their employment in biomedical applications is rapidly increasing [21]. The surgical environment should be taken into account too, in order to select the appropriate sensor typology. In case of electromagnetic interference, such as during an MRI-guided intervention, or in case of a metallic object in the surgical area (e.g., a dental implant) an optical sensor should be preferred compared to an electrical one in light of electromagnetic inertness. On the contrary, in case of high temperatures and significant vibrations, an electrical-based sensor is to be preferred. Nonetheless, specific articles regarding the application of these technologies in the field of maxillofacial surgery have not been retrieved (Table 6).

### 3.2. Sensorized Tools for Contact Forces Detection: Dentistry and Maxillofacial Applications

We retrieved a shortage of manuscripts regarding sensorized tools for contact force detection in dentistry and maxillofacial applications [24]. Nonetheless, this field should be expanded considering that maxillofacial surgery interventions are complex due to different types of anatomical structure in restricted surgical fields, each of them requiring specific procedures and appropriate skills. Moreover, esthetical appearance is essential for patients and must be considered to evaluate postoperative results. Nowadays, maxillofacial procedures are performed manually for the majority, using different tools (e.g., pincers, drill, saw) -with limitations related to the intervention’s duration (often more than eight hours), tiredness of surgeon, meanwhile requiring high accuracy in bone positioning—trying to minimize tissue and vessels’ damages [22]. In the orthodontics field, an open issue is to detect and measure loads and forces applied during treatments performed with different tools in order to prevent damage. Hence, the requirement to design and develop sensorized surgical tools able to measure tool–tissue contact forces and suitable to act synergically with a haptic device that provides the tactile sensation to the user. A customized measuring system, able to reveal forces in a range between 0.1 and 2 N, was designed by Mencattelli [24]. The sensing unit is composed of six load cells each coupled with a strain gauge. 

### 3.3. Main Actuation Principles for Haptic Feedback: Dentistry and Maxillofacial Applications

Haptic devices, able to supply haptic feedback to the user, can be classified into two main families: grounded devices, with the base fixed to the ground, and wearable devices [25]. In the following surgical applications, commercial grounded interfaces are mainly employed with only some exceptions regarding novel design wearable interfaces. Grounded devices can display a vast range of forces at the expense of portability, high cost, and bulk. On the contrary, portable devices’ strengths are wearability, low weight and volume, and reduced form factor, despite the haptic stimuli provided being still limited to vibrations with a consequent inability in reproducing complex contact interactions. To enhance the wearability of haptic interfaces, studies are focused on relocating the grounding of the system closer to the point of actuation of the stimulus although the kinesthetic component is reduced, preserving the cutaneous impulse. In the borderline case, when the grounding part is coincident with the stimulus application point, the system provides only cutaneous feedback anyway without compromising tactile discrimination [26]. A possible classification of wearable haptic devices is based on haptic systems’ mechanical properties: Degrees of Freedom (DoF) of the end effector, workspace, precision of forces and position rendering (describes the reproducibility of a specific action), resolution of forces and bandwidth. A fully actuated haptic device is able to render 3-Dof forces/torques, taking into account that increasing the DoF number leads to a consequent increase in the design’s complexity. Haptic devices can be categorized based on the action’s area (fingertip or whole hand) and subdivided for the type of cutaneous cues they are able to provide: normal indentation, lateral skin stretch, and vibration (vibrotactile stimuli due to vibrotactile actuators) for fingertip systems, kinesthetic or vibration for whole hand systems. In fingertips systems based on normal indentation, actuation could be obtained using a moving system in contact with the finger pulp [27], pin arrays—with different pin diameters and pin spacing, usually moved by DC motors, servo motors, shape memory alloy (SMA) actuators, pneumatic actuators, dielectric elastomer actuators [28]—or pneumatic systems (jets and balloon-based systems). The actuation is usually due to DC motors, servomotors, or SMA actuators. Whole hand devices (also called hand exoskeletons) can provide cutaneous and kinesthetic stimuli to the entire hand. These systems employ actuation technologies used for fingertip devices but differently, they can provide kinesthetic and/or pressure and/or vibration stimuli to the user. Open issues involve kinematic coupling between the wearer and exoskeleton joints without meddling with the wearer’s motions and low wearability of systems providing kinesthetic feedback. As a solution, vibrotactile actuators with small form factors and low weight are employed to supply vibrotactile feedback instead of the kinesthetic one [24]. 

Haptic interfaces are widely used in dental and maxillofacial surgery, especially for digital simulation purposes. A dental implant surgery simulator (DISS), based on the haptic device Omega.6 (Force Dimension, Nyon, Switzerland) and CHAI3D software(Artificial Intelligence Laboratory at Stanford University, USA), is proposed in [29]. Driller diameter and drill speed are variable during simulation, conducted on patient-specific virtual models. Users wear 3D glasses to receive visual feedback too. A voxel-based approach to reconstruct oral tissues and an analytical drilling force model for haptic simulation of micro-implants procedure are proposed in [30]. The aim is to train orthodontists in this common procedure minimizing risks to patients’ tooth roots and providing real-time force feedback from different tissue layers. Surgeons must be careful to apply appropriate forces performing procedures such as Bilateral Sagittal Split Osteotomy (BSSO), the most common mandibular intervention, which requires cutting the mandible through a burr and oscillating saw. BSSO simulator [31] provides visual feedback to the user with Oculus Rift HMD and force feedback through Geomagic Touch haptic device. Prototyping of interactive virtual environments is implemented with the interactive medical simulation toolkit iMSTK. Wu et al. [32] implemented a multimodal (visual, touch, and sound) VR training system for Le-Fort I osteotomy with virtual 3D models of anatomic structures reconstructed from CT patient data. The hardware architecture is composed of a 3D Display 300 (SenseGraphics, Sion, Switzerland), a 2D LCD monitor, and a force feedback haptic device Omega.6. VS2008 is the Integrated Development Environment software (Microsoft, Washington, DC, USA) and CHAI3D (Artificial Intelligence Laboratory at Stanford University, Stanford, CA, USA) allows the communication with the haptic manipulator. Omega.6 does not provide torque feedback and the maximum force detected during simulation is only 12 N, lower than typical forces commonly used, thus a novel haptic device is required. Common procedures in maxillofacial surgery such as open reduction and palate fixation can be performed using the digital VR simulator proposed by Zhang et al. [33]. In cranio-maxillofacial surgery, an important issue is restoring normal skeletal anatomy in patients with complex and massive traumas. Regarding the reduction of maxillary fractures and facial traumas, novel training for visuo-haptic simulators has been developed [34,35]. In [34], a Geomagic Phantom (3D Systems, Rock Hill, SC, USA) desktop haptic device (providing 6-DOF input and 3-DOF force output) and CHAI 3D software allow it to operate in the 3D virtual environment with haptic sensations. A Lenovo ThinkPad workstation (Lenovo, Beijing, China) is used to simulate the immersive scenario. To solve the issue of low maximum output force [7.9 N] related to Geomagic, authors set different levels of force feedback to allow users to feel changes in motions [35]. The authors highlighted better user experiences, intuitiveness, and postoperative results of Computer-Assisted Surgical (CAS) systems compared to computer-aided design (CAD) systems used in bone fracture reduction, providing a sense of touch to users. Additionally, it is proved that appropriate preoperative planning systems cause reduced operating time and morbidity in patients who need this operation [36]. Olsson et al. proposed a new surgery preoperative planning system with visuo-haptic feedback regarding bone fragments reconstruction [37]. Medellin-Castillo et al. deal with the topic of cephalometry [38,39]. They demonstrated that 3D cephalometry significantly reduces landmarking errors compared to 2D methods and proposed a haptic VR system involving cephalometry analysis reducing bias versus the traditional approach. It should be kept in mind that force feedback on different devices does not influence the simulation’s performances in a significant way as Bugdadi’s work assessed [40]. Despite the growing relevance of haptic simulators due to several advantages in oral and maxillofacial application these devices are underused too, probably because of the lack of scientific studies reported on in the literature [41]. The main future challenge will be to provide appropriate real-time haptic sensations during simulation therefore the research should be conducted in this direction. 

### 3.4. Integrated Platforms for Surgery and Telemedicine: Dentistry and Maxillofacial Applications

Computer-assisted surgery had boundless progress in a short time, realizing improvements for patients and surgeons thanks to the increasing use of robotic platforms for surgery [1]. In the following sections, main robotic platforms have been reviewed, including mannequin simulators (surgical tool–target physical interactions) with haptic feedback for dentistry-maxillofacial surgery. Haptic devices let a user interact with a real remote object/structure or with a computer-generated environment, providing a sense of tangibility—called haptic feedback [42]—stocked by the device’s actuators. A haptic device is a robot with a different number of degrees of freedom (DOF)—depending on structure and design—located at a control station and moved directly by the operator [43] in order to give feedback on movements and forces imposed by the user himself. The haptic tool can provide tactile feedback due to mechanoreceptors, kinesthetic feedback due to muscle tensions, or rarely thermal feedback. For years, numerous commercial grounded haptic devices have been proposed trying to satisfy the demanding requests in terms of huge workspace, kinematic isotropy, and force/torque provided. Some of the commercial haptic devices usually employed for surgical applications have been developed by Force Dimension (Omega 3, Omega 6, and Omega 7 devices able to provide force feedback with 3, 6, and 6 + 1 DOF, respectively), SensAble Technologies (Phantom Omni, Phantom Desktop supplying force feedback with 3/6 DOF), Moog FCS Robotic (HapticMaster with 3 DOF), Novint (Novint Falcon™ with 3 DOF) and Entact Robotics (Entact W5D with 5 DOF) [42]. The main current limits related to these technologies are the reduced workspaces, different from conventional surgery, and lack of specificity. Given these limitations, surgeons must adapt gestures and movements to the instrument’s structure, the direct line of sight between surgeon and device could induce distractions as well as holding a rigid and bulky object can lead to surgical errors.

Surgical simulators were introduced for the first time in the 1990s with the aim to simulate immersive and specific scenarios and simplify training for complex surgical procedures [41]. Novel simulators employ multimodal (visual, touch, sound) virtual reality (VR) in order to improve training with countless advantages. VR simulators are cost-effective, easy to use, can automatically assess surgeons’ performances, and provide objective feedback measures as well as enhance strength, speed, and dexterity [31]. Due to the complexity of oral anatomy, VR gains a preeminent role in operations planning and surgical training. Usually, preoperative planning or surgical training starts with the development of a three-dimensional (3D) anatomical model, obtained by the patient themself with imaging techniques, using software technologies. Next to the visual domain, tactile sensations such as vibrotactile or pressure stimuli, provided by a haptic interface, can be integrated to obtain an immersive and realistic surgical scenario in order to let the users feel and touch different structures. A 3D virtual environment is essential to prevent setbacks in oral restorative dentistry, prosthetic dentistry, or maxillofacial surgery as well as to increase novices’ performances during training.

Moreover, VR simulators allow us to overcome the limits of traditional simulation such as the cost and availability of animal or cadaveric models and time-consuming practice under the supervision of experienced surgeons as well as the reduced biomimetic of physical simulators. These evaluations contributed to the growth of mannequin and digital VR simulators. Incorporating haptic feedback into VR training systems consent to obtain better results in terms of performance accuracy, safety, skills acquisition, and learning speed [5]. The main digital/physical simulators presented in our overview have been reported and are summarized in Table 7 based on the surgical application field. 

The first robotic-assisted maxillofacial intervention in animal models was executed in 2003. Transoral Robotic Surgery (TORS) became a disruptive surgical technique with the FDA approval of the Da Vinci Surgical System for TORS (Intuitive Surgical Inc., Sunnyvale, CA, USA) in 2009. The effectiveness of these systems was evaluated over several years in terms of safety, functional outcomes, oncological outcomes, reduced postoperative complications, and cost. Limitations include limited accessibility of bulky instruments, initial long setup, long operative times, and lack of force feedback and tactile perception of tissues [1]. This robot’s first generation was modified in 2015 with the introduction of FLEX Robotic System (Medrobotics Inc., Raynham, MA, USA) a robotic platform with higher maneuverability. Nowadays robotic surgery systems are commonly employed in head and neck neoplasm resection, cleft lip and palate, fracture reduction, and OSAS (the most common type of sleep apnea) treatments [3]. However, the lack of haptic and force feedback in each commercial existing system must be noticed. Novel systems should be designed to allow integration with the most common commercial robotic platforms providing tactile sensations to the user. For instance, Pacchierotti [44] implemented a cutaneous feedback interface integrated on an Intuitive Surgical da Vinci Standard, able to measure contact deformations, in the end effector of a surgical instrument. Haptic feelings are provided to the user by fingertip deformations with a significant improvement in palpation performances.

Next to master–slave systems, such as the Da Vinci Surgical System, in which the surgeon interacts with the patient through a console (master) that enables surgical robotic tools, the employment of cooperatively controlled robots is emerging in the implant surgery field. Cooperatively controlled systems are typically grounded platforms, with a 6-axis robotic arm; the user can interact with them by manual control. The surgeon has real-time control of the surgical tool while the robot offers assistance during the surgical tasks execution improving performance, precision, and providing force constraints. This type of platform can also provide surgeon’s motion scaling or tremor suppression thanks to a force/torque sensor usually located between the end effector and the cinematic chain. 

Three-dimensional printed models are widely employed in physical surgical simulators for dental and maxillofacial applications enabling surgeons to understand anatomy/pathology and practice intricate tasks, too [45]. Syed et al. [46] proposed a multi-arm medical platform with a 6-DoF surgical manipulator and haptic device with force feedback (Omega.6 haptic device) able to improve surgical accuracy during maxillofacial teleoperations. A pressure commercial sensor (force–torque sensor IFS50M31A25-I25) is installed on the manipulator end effector to detect collisions and transfer the intensity value to a haptic device. A VR haptic platform for endoscopic sinus and skull base surgeries was presented by Kim [47]. The platform’s architecture is composed of two Geomagic Touch X haptic devices to supply haptic feedback, a monitor for visual feedback, and an endoscope device. The simulator can allow different procedures from maxillary sinus antrostomy, ethmoidectomy, and frontostomy to endoscopic endonasal trans-sphenoidal and transclival approaches [47]. As stated by the authors, the main limit is that the simulated artery does not bleed. It is useful for training based on reported results and, in the future, the assessment system will be able to perform a behavior-based task analysis based on the simulation performance of the trainee, adjusted according to their level of experience [47]. Moreover, regarding the application of virtual reality, Girod et al., (2016) presented a newly developed haptic system (Geomagic Touch, 3D Systems, Rock Hill, SC, USA). The system was tested by ten surgeons on 3 different clinical cases of mandibular fracture reduction. By comparing standard landmarks and linear and angular measurements between the simulated results and the actual surgical outcome no difference was found between the virtual simulator and actual postoperative outcomes [35]. Regarding dental surgery, a patient-specific haptic drilling simulator based on virtual reality for dental implant surgery was developed, as reported in paragraph 3.3. The dental implant surgery simulator (DISS) was evaluated by 30 novice surgeons with positive preliminary results based on subjective scales [29].

## 4. Discussion

In this study, a comprehensive overview of the newest haptic and remote technologies employed in dentistry and maxillofacial surgery was conducted. Given the results of our overview, tactile sensors for instrumented tools [17,18,19,20,21], sensorized tools for contact forces detection [22,24], haptic interfaces for remote tactile feedback [23,25,26,27,28,29,30,32,34,35,36,37,38,40,41,42,43,44,45,46,47], and integrated platforms for surgery and telemedicine [29,31,35,37,39,40,43], are four major categories of novel technologies applied in dentistry and maxillofacial surgery. A major concern at the actual state of the art is the absence of randomized control trials and the presence of few case reports [23,31,37,39,45] and retrospective cohorts [36] with a prevalence of experimental studies [22,24,26,27,28,29,30,32,33,34,35,38,40,43,44,46,47].

Based on the results of our overview, many efforts have been made to overcome the technological limits of CAS surgery. Nonetheless, a research gap between dental/maxillofacial surgery and other surgical specialties such as endovascular treatments, laparoscopic surgery, and microsurgery needs to be stressed [51,52,53]. Furthermore, this work is intended to resemble technological trends in haptic transducing mechanisms in maxillofacial and dental surgery applications. Nowadays, single-point tactile sensors are combined in a certain way, to obtain a flat tactile sensor array [54]. Sensing units are arranged in a matrix with M × N dimensions (M is the number of rows, and N is the number of columns of sensing units). Tactile sensor arrays allow a higher contact area with the target as well as the ability to detect stimuli from different directions. Consequently, the obtainable spatial resolution is better than single-point tactile sensors. Furthermore, tactile sensors could be used to obtain the mechanical properties of tissues, too. In fact, surgeons typically need magnitude or force feedback while performing different tasks. Despite the major adoption in each surgical field of electrical-based tactile sensors (e.g., piezoresistive and piezoelectric tactile sensors) during the years, over the last decade, the adoption of optical-based tactile sensors has been growing. A preeminent adoption and development of optical and FBGs-based sensors/tools has been highlighted especially in microsurgery, endovascular procedures [55], MIS- [20] and MRI-guided interventions because of unique properties essential in biomedical applications. These properties include high versatility, easily embedded in different materials and tight volumes, flexibility, high sensitivity, electromagnetic compatibility, and biocompatibility with human tissues [56]. Moreover, optical sensors can be easily organized in arrays with different geometries in order to detect information from the paths of interest or sense force distributions. Noteworthy drawbacks of optical fiber-based sensors are the higher costs and design complexity in comparison with the electrical ones. The previous limitations could be overcome by increasing targeted research and studies. The employment of optical and FBGs-based tactile sensors in novel tools specially designed for dental/maxillofacial surgery purposes, in light of the excellent technological results exposed in the literature [17,18,19,20,21], could be an innovative and disruptive idea to enhance surgical performances simultaneously reducing drawbacks.

Next to conventional electrical and optical sensing technologies, a novel approach based on microfluidic sensors looks promising [57]. Microfluidic sensors are realized by embedding a liquid-state metal into elastic microchannels. Forces, due to mechanical stimuli, can be detected considering the flow of liquid, through specific microchannels, which define the sensor output. The applied force causes a variation in the electrical/optical properties of the fluid. PDMS (Polydimethylsiloxane) is the elastomeric material most used in microfluidics in light of its high flexibility, biocompatibility, low cost, and resilience. Examples of microfluidics tactile sensors are impedance-based sensors (in which the impedance variations in electrolyte volume near the electrodes are a function of the mechanical deformation due to the load), resistive-based sensors (the elastomeric deformation induces a change in the microchannels resistance) and capacitive-based sensors. Despite several open related points—such as the long-term instability of the microfluidic sensor, the low electronic compatibility, and the breakdown of the elastomeric structure—the benefits of this emerging technology could revolutionize the tactile sensing methodology in surgery [58].

Tactile sensors for instrumented tools (TS-IT) and sensorized tools for contact forces detection (ST-CFD), by monitoring and controlling the forces being applied during surgical procedures, can be useful in open surgery, MIS and CAS [42]. In maxillofacial surgery, the delicate anatomy and close proximity to important structures such as nerves and blood vessels require a high degree of precision [59,60,61]. TS-IT and ST-CFD can provide real-time feedback on the forces being applied during surgery, allowing surgeons to adjust their technique to avoid damaging the fines abovementioned structures. Surgeons can achieve better outcomes for the patient and avoid applying excessive force that can lead to complications such as bone fractures or neurotomy. Moreover, these tools can help to standardize surgical techniques and outcomes. By providing objective measurements of the forces being applied, surgeons can compare their techniques, allowing for better quality control and constant improvement.

Virtual reality, by creating a computer-generated image that mimics the real environment, has the potential to revolutionize teaching and self-learning [35]. Students can be educated outside the hospital and still have access to a high level of training by using hands and tools in a simulated environment to gain experience and understanding of medical procedures before entering the operating room [47]. In the near future, virtual reality simulation will likely become an essential part of medical education. Therefore, a strict evaluation by the scientific community about these technologies is mandatory [29]. Advantages are the potential for flexibility and cost-effectiveness compared to laboratory training methods requiring cadavers, whereas the main disadvantages are that they cannot replace real-life experience.

In the future, a great contribution can be expected by artificial intelligence. The use of machine learning algorithms to enhance tactile perception in maxillofacial surgery is an exciting area of research, with significant potential for improving the accuracy and effectiveness of surgical procedures: with the emerging request of telemedicine from hospitals without maxillofacial specialists, we hope that haptic sensors will make it possible to visit patients remotely, and we also hypothesize the possibility of performing interventions remotely. By treating tactile elements as image pixels, machine learning algorithms can be used to analyze and interpret tactile information in real time, providing surgeons with valuable information about the patient’s anatomy and the surgical site. As more research is conducted in this area, we can expect to see the development of increasingly sophisticated algorithms that can provide surgeons with valuable real-time information, ultimately leading to better outcomes for patients [54]. Integration of different technologies embedded with the help of artificial intelligence can be hypothesized as the next future of maxillofacial surgery practice and teaching.

## 5. Conclusions

The limits of the present work are mainly related to the low quality of the available literature. Nonetheless, we have summarized and evaluated current advantages, drawbacks, and design choices of new technologies for each field of application in maxillofacial surgery. Tactile sensors for instrumented tools, sensorized tools for contact force detection, haptic interfaces for remote tactile feedback, and integrated platforms for surgery and telemedicine are technologies currently available and increasingly applied in maxillofacial surgery. Future works, especially RCT, should be carried on in these fields to define the future guidelines of treatment. 

## Figures and Tables

**Table 1:** General characteristics, study population, and relevant findings of each study are included in the present overview.

First Author, Year [Reference]	Country	Type and Grade ^#^	Field	Subjects	Category	Original Technology	Conclusions
Arikatla 2018 [31]	USA/India	CR (4/7)	Orthognatic surgery	1	Integrated platforms	NA	In the future, we will employ the methods proposed in this paper to build a prototype that simulates the full procedure of the BSSO.
Bandari 2016 [17]	Canada	Review (10/12)	MIS and RMIS	NA	TS-IT	NA	There is a relatively small number of recent developments of hybrid sensors for MIS and RMIS.
Bugdadi 2018 [40]	Canada	Exp (6/6)	Neurosurgery	6 *	Haptic feedback; Integrated platforms	NA	To maximize realism of the training experience, educators employing virtual reality simulators may find it useful to assess expert opinion before choosing a force feedback device.
Chen 2018 [29]	China	Exp (6/6)	Dentistry	30 **	Haptic feedback; Integrated platforms	DISS	The DISS may provide an alternative training method for the surgeons to enhance their dental implant surgical skills and experiences.
Dahiya 2010 [18]	Italy	Review (7/12)	Translational	NA	TS-IT	NA	Much work needs to be conducted at the system level before artificial touch can be used in a real-world environment.
Frediani 2014 [28]	UK/Italy	Exp (5/6)	Translational	NR	Haptic feedback	bubble like HC-DEA	A novel tactile display able to simulate contact with virtual soft bodies via soft interfaces, offering low weight, no acoustic noise, no heating, scalability, and low power consumption.
Giri 2021 [42]	Canada	Review (5/12)	Translational	NA	Haptic feedback	NA	The medicinal world is still skeptical about the usage of haptic devices in surgeries and training.
Girod 2015 [35]	USA	Exp (5/6)	Maxillofacial	10 **	Haptic feedback; Integrated platforms	Geomagic Touch, 3D Systems	Our advanced haptic surgical planning system enabled surgeons to simulate mandibular fracture repair more accurately and with a better user experience than a CAD system.
Hoshyarmanesh 2021 [43]	Canada	Exp (5/6)	MIS and RMIS	NR	Haptic feedback; Integrated platforms	neuroArmPLUS	This manuscript describes the engineering principles behind the design and development of a microsurgery-specific haptic interface. Establishing its clinical use is outside the scope of this work.
Kim 2020 [47]	Korea	Exp (4/6)	FESS	NR	Haptic feedback; Integrated platforms	VR haptic platform	VR haptic simulators can improve the skill and confidence of surgical trainees by allowing them to accrue experience in various tasks under different conditions.
Liang 2018 [20]	China/Canada	Review (5/12)	Translational	NA	TS-IT	NA	Significant improvements have been achieved in addressing numerous aspects of designing and developing multicomponent opto-electric force sensing systems.
Liu 2012 [22]	China	Exp (4/6)	Maxillofacial	NA	ST-CFD	Control System for CMF Robot	The repeatability accuracy experiment showed that the movement of the robot is smooth, stable, and safe.
Lo presti 2020 [21]	Spain/Portugal/Italy/Usa	Review (6/12)	Translational	NA	TS-IT	NA	The considerable amount of attention given to FBGs in scientific papers and the growing market interest regarding their applications in medicine underline the strong interest in fulfilling the gap between research and clinical practice.
Maliha 2018 [41]	USA	Review (10/12)	Maxillofacial	NA	Haptic feedback	NA	Although seemingly beneficial to the trainee in maxillofacial surgery, simulation in education in this field is an underused commodity because of the significant lack of scientific and validated study designs reported on in the literature thus far.
Medellìn-Castillo 2016 [38]	Mexico/UK	CS (4/10)	Maxillofacial	5	Haptic feedback	NA	A haptic-enabled approach is feasible in 2D, 2^1⁄2^D, and 3D environments and benefits were obtained in the reduction of measurement errors, lower variability, and reduced task completion times.
Mencattelli 2014 [24]	Italy	CR (4/8)	Dentistry	2	ST-CFD	Measuring platform	This measuring system allows measurements of 6 orthodontic forces exerted by any orthodontic device.
Nicot 2017 [23]	France	CR (4/7)	Maxillofacial	1	Haptic feedback	NA	The description of defects through haptic 3D printed models may be the next step in the provision of parental information.
Nilsson 2020 [36]	Denmark/Sweden	CS (3/10)	Maxillofacial	12	Haptic feedback	NA	In this study, we present an in-house haptic-assisted planning tool with high usability that can be used for preoperative planning and evaluation of complex mandible fractures.
Olsson 2013 [37]	Sweden	CR (5/6)	Maxillofacial	1	Haptic feedback	NA	Preliminary testing with one surgeon indicates that our haptic planning system has the potential to become a powerful tool that with little training allows a surgeon to complete a complex CMF surgery plan in a short amount of time.
Pacchierotti 2015a [26]	Italy	Exp (5/6)	Translational	15 ***	Haptic feedback	3-DoF cutaneous haptic device	Cutaneous feedback showed better performance than employing no force feedback at all, but, as expected, it was outperformed by full haptic feedback provided by grounded haptic interfaces.
Pacchierotti 2015b [44]	Italy/USA	Exp (5/6)	Translational	18 ***	Haptic feedback	SynTouch BioTac	Subjects who used a dragging strategy achieved even better results with cutaneous feedback of fingertip vibrations. Subjects also highly preferred conditions providing cutaneous feedback over the one without any haptic feedback.
Pacchierotti 2017 [25]	France/Italy	Review (6/12)	Translational	NA	Haptic feedback	NA	The “wearables” technology trend will continue to play a strong role in pushing haptics forward in the coming decade.
Prattichizzo 2013 [27]	Italy	Exp (5/6)	Translational	14 ***	Haptic feedback	Novel 3-DoF wearable display	In comparison to similar existing cutaneous devices, this one has three actuated degrees of freedom and it is able to simulate a contact force with general direction at the fingertip.
Saccomandi 2014 [19]	Italy	Review (7/12)	Translational	NA	TS-IT	NA	The growing and continuous research in the field of tactile sensing for biomedical applications will go towards the fusion of many technologies, aiming to enhance the pros of each technique.
Shujaat 2021 [45]	Belgium/Sweden	CR (4/7)	Orthognatic surgery	1	Haptic feedback	NA	Our findings provide evidence on the anatomical and haptic quality 3D models with various printers which may guide physicians and trainees to select a certain printer and material depending on the task at hand.
Syed 2013 [46]	China	Exp (4/6)	Maxillofacial	NR	Haptic feedback	6-DOF	The repeatability Exp results show that the movement of the manipulator under satisfactory boundaries, which is suitable and fulfills the needs of the surgery.
Wu 2014 [32]	China	Exp (4/6)	Maxillofacial	25 **	Haptic feedback	VR-MFS	The VR-MFS provides an efficient and cost-effective way to train maxillofacial novices.
Zaragoza-Siqueiros 2019 [39]	Mexico/UK	CR (4/7)	Orthognatic surgery	6 **	Haptic feedback; Integrated platforms	OSSys	The proposed system integrates the four main stages of the traditional OGS planning process: clinical facial analysis, cephalometric analysis, model surgery, and surgical template generation.
Zhang 2015 [34]	China	Exp (4/6)	Maxillofacial	NR	Haptic feedback	Digital VR simulator	This system provides training to medical students, and can also be used in preoperative planning.
Zhang 2021 [33]	China	Exp (5/6)	Maxillofacial	10 **	Haptic feedback		There are still a lot of technical challenges in the development of virtual surgery.
Zheng 2012 [30]	China	Exp (4/6)	Dentistry	NR	Haptic feedback		This paper presents an effective framework to simulate the pilot-drilling procedure of the micro-implants surgery.

Abbreviations: BSSO (Bilateral Sagittal Split Osteotomy); CAD (Computer-Aided Design); CMF (Cranio Maxillofacial); CR (Case Report); CS (Case Series); DISS (Dental Implant Surgery Simulator); FESS (Fiberoptic Endoscopic Sinus Surgery); HC-DEA (hydrostatically coupled dielectric elastomer actuators); MIS (Minimally Invasive Surgery); NA (Not Aplicable); NR (Not Reported); OGS (Orthognatic Surgery); RMIS (Robotic Minimally Invasive Surgery); ST-CFD (Sensorized tools for contact forces detection); TS-IT (Tactile Sensors for Instrumented Tools); VR-MFS (Virtual Reality Maxillofacial Surgery). Legend: * Neuro Touch expert’s Surgeons; ** resident trainee in oral/maxillofacial surgery or plastic surgery with no prior experience with haptic systems; *** different types of volunteers; **^#^** the appropriate JBI’s Clinical Appraisal Tool for each different type of included studies was applied [48,49]. A scale for the quality assessment of narrative review articles (SANRA) was also used when appropriate [50].

**Table 2:** JBI CR evaluations.

	Olsson				Nicot				Shujaat				Arikatla				Zaragoza				Mencattelli			
	Yes	No	Unclear	Not applicable	Yes	No	Unclear	Not applicable	Yes	No	Unclear	Not applicable	Yes	No	Unclear	Not applicable	Yes	No	Unclear	Not applicable	Yes	No	Unclear	Not applicable
Were the patient’s demographic characteristics clearly described?	1	□	□	□	1				1				1				1				0	1		
Was the patient’s history clearly described and presented as a timeline?	0	□	□	□	0				0				0				0				0	1		
Was the current clinical condition of the patient on presentation clearly described?	1	□	□	□	1				0				0				0				1			
Were diagnostic tests or assessment methods and the results clearly described?	□	□	□	1	1				1				1				1				1			
Was the intervention(s) or treatment procedure(s) clearly described?	1	□	□	□				1	1				1				1				1			
Was the post-intervention clinical condition clearly described?	1	□	□	□	0				0				0				0				0	1		
Were adverse events (harms) or unanticipated events identified and described?	□	□	□	1	0							1				1				1		1		
Does the case report provide takeaway lessons?	1	□	□	□	1				1				1				1				1			
Total	5/6				4/7				4/7				4/7				4/7				4/8			

**Table 3:** JBI CS evaluations.

	Nilsson				Medellìn-Castillo			
	Yes	No	Unclear	Not Applicable	Yes	No	Unclear	Not Applicable
Were there clear criteria for inclusion in the case series?	□	□	1	□			1	
Was the condition measured in a standard, reliable way for all participants included in the case series?	□	□	1	□	1			
Were valid methods used for identification of the condition for all participants included in the case series?	1	□	□	□	1			
Did the case series have consecutive inclusion of participants?	1	□	□	□			1	
Did the case series have complete inclusion of participants?	□	□	1	□			1	
Was there clear reporting of the demographics of the participants in the study?	□	1	□	□		1		
Was there clear reporting of clinical information of the participants?	□	1	□	□		1		
Were the outcomes or follow-up results of cases clearly reported?	□	1	□	□	1			
Was there clear reporting of the presenting site(s)/clinic(s) demographic information?	□	1	□	□		1		
Was statistical analysis appropriate?	1	□	□	□	1			
	3/10				4/10			

**Table 4:** JBI Exp evaluations. * more than 20 references; ^§^ expert panel; ^$^ refer to completeness of Section 2; ^£^ refer to general quality and completeness of the manuscript.

	Bugdadi				Chen				Frediani				Girod				Hoshyarmanesh				Kim				Liu				Pacchierotti 2015a				Pacchierotti 2015b				Prattichizzo 2013				Syed				Wu				Zhang 2015				Zhang 2021				Zheng 2012			
	Yes	No	Unclear	Not applicable	Yes	No	Unclear	Not applicable	Yes	No	Unclear	Not applicable	Yes	No	Unclear	Not applicable	Yes	No	Unclear	Not applicable	Yes	No	Unclear	Not applicable	Yes	No	Unclear	Not applicable	Yes	No	Unclear	Not applicable	Yes	No	Unclear	Not applicable	Yes	No	Unclear	Not applicable	Yes	No	Unclear	Not applicable	Yes	No	Unclear	Not applicable	Yes	No	Unclear	Not applicable	Yes	No	Unclear	Not applicable	Yes	No	Unclear	Not applicable
Is the source of the opinion clearly identified? ^£^	1	□	□	□	1	□	□	□	1	□	□	□	1	□	□	□	1	□	□	□	1	□	□	□	1	□	□	□	1	□	□	□	1	□	□	□	1	□	□	□	1	□	□	□	1	□	□	□	1	□	□	□	1	□	□	□	1	□	□	□
Does the source of opinion have standing in the field of expertise? ^§^	1	□	□	□	1	□	□	□	□	1	□	□	1	□	□	□	□	1	□	□	□	1	□	□	□	1	□	□	□	1	□	□	□	1	□	□	□	1	□	□	□	1	□	□	1	□	□	□	□	□	□	□	1	□	□	□	□	1	□	□
Are the interests of the relevant population the central focus of the opinion?	1	□	□	□	1	□	□	□	1	□	□	□	1	□	□	□	1	□	□	□	1	□	□	□	1	□	□	□	1	□	□	□	1	□	□	□	1	□	□	□	1	□	□	□	1	□	□	□	1	□	□	□	1	□	□	□	1	□	□	□
Is the stated position the result of an analytical process, and is there logic in the opinion expressed? ^$^	1	□	□	□	1	□	□	□	1	□	□	□	1	□	□	□	1	□	□	□	1	□	□	□	1	□	□	□	1	□	□	□	1	□	□	□	1	□	□	□	1	□	□	□	1	□	□	□	1	□	□	□	1	□	□	□	1	□	□	□
Is there reference to the extant literature? *	1	□	□	□	1	□	□	□	1	□	□	□	□	1	□	□	1	□	□	□	□	1	□	□	□	1	□	□	1	□	□	□	□	1	□	□	1	□	□	□	□	1	□	□	1	□	□	□	□	□	□	□	1	□	□	□	1	□	□	□
Is any incongruence with the literature/sources logically defended?	1	□	□	□	1	□	□	□	1	□	□	□	1	□	□	□	1	□	□	□	1	□	□	□	1	□	□	□	1	□	□	□	1	□	□	□	1	□	□	□	1	□	□	□	1	□	□	□	1	□	□	□	□	1	□	□	□	1	□	□
Total	6/6				6/6				5/6				5/6				5/6				4/6				4/6				5/6				5/6				5/6				4/6				6/6				4/6				5/6				4/6			

**Table 5:** SANRA evaluations.

	Bandari	Dahiya	Giri	Liang	Lo presti	Maliha	Pacchierotti	Saccomandi
(1) Justification of the article’s importance for the readership. The importance is not justified. 0 The importance is alluded to, but not explicitly justified. The importance is explicitly justified.	2	2	1	1	2	1	2	2
(2) Statement of concrete aims or formulation of questions. No aims or questions are formulated. 0 Aims are formulated generally but not concretely or in terms of clear questions. One or more concrete aims or questions are formulated.	2	1	1	0	1	2	0	1
(3) Description of the literature search. The search strategy is not presented. The literature search is described briefly. The literature search is described in detail, including search terms and inclusion criteria	2	0	0	0	0	2	0	0
(4) Referencing Key statements are not supported by references. 0 The referencing of key statements is inconsistent. 1 Key statements are supported by references. 2	2	2	2	2	2	2	2	2
(5) Scientific reasoning (e.g., incorporation of appropriate evidence, such as RCTs in clinical medicine). The article’s point is not based on appropriate arguments. 0 Appropriate evidence is introduced selectively. 1 Appropriate evidence is generally present. 2	0	0	0	0	0	2	0	0
(6) Appropriate presentation of data (e.g., absolute vs. relative risk; effect sizes without confidence intervals). Data are presented inadequately. 0 Data are often not presented in the most appropriate way. 1 Relevant outcome data are generally presented appropriately. 2	2	2	1	2	1	1	2	2
Total	10/12	7/12	5/12	5/12	6/12	10/12	6/12	7/12

**Table 6:** Summary of main tactile sensing transduction mechanisms currently employed in biomedical applications with advantages and disadvantages.

	*Sensing Principle*	*Advantages*	*Disavantages*
*Piezoresistors*	Resistance variation	Excellent spatial resolution High sensivity Low cost	Low frequency response Low repeatability
*Capacitive sensors*	Capacitance variation due to a mechanical force or moment	Temperature independent High spatial resolution High sensivity	Crosstalk between elements Susceptible to noise Stray capacitance hysteresis
*Inductive sensors*	Magnetic coupling variation due to a mechanical force or moment	High dynamic range High sensivity Linear output	Low frequency response Poor reliability
*Strain gauges sensors*	Change in resistance because of shape deformation	High spatial resolution Easy design Low cost	Largge hysteresis Non-linear response
*Piezoelectric sensors*	Strain polarization	High accuracy High dynamic range High frequency response	Low spatial resolution High temperature susceptibility
*Optical sensors*	Change in wavelenght	High spatial resolution and sensivity Electomagnetically inert	High temperature susceptibility High cost Size limitations

**Table 7:** Simulators with haptic feedback retrieved in the present overview: no wearable devices were retrieved.

	First Author, Year [Reference]	Medical Procedure	Type of Simulator	Haptic Device	Simulation’s Software
Dentistry	Chen 2018 [29]	DISS	Digital	Omega.6	CHAI3D 1.1
	Zheng 2012 [30]	Micro-implants surgery	Digital	Phantom Desktop	-
Maxillofacial Surgery	Arikatla 2018 [31]	BSSO	Digital	Geomagic Touch	toolkit-iMSTK
	Wu 2014 [32]	Le-Fort I osteotomy	Digital	Omega.6	VS2008 CHAI3D
	Zhang 2015 [34]	Reduction of maxillary fractures	Digital	Geomagic Phantom	CHAI3D
	Zhang 2021 [33]	Open reduction and plate fixation	Digital	Geomagic Phantom desktop X	OpenGL
	Kim 2020 [47]	Endoscopic sinus and skull base surgeries	Physical	Geomagic Touch X	Unity3D

Abbreviations: DIS (dental implant surgery simulator); BSSO (bilateral sagittal split osteotomy).

## Data Availability

Not applicable.
